# Supplementary material for: Integrating an addiction team into the management of patients transplanted for alcohol-associated liver disease reduces the risk of severe relapse
Source: JHEP Rep. 2023 Jul 30;5(10):100832. doi: 10.1016/j.jhepr.2023.100832 (PMC10480527; doi:10.1016/j.jhepr.2023.100832)

Supplementary materials

Integrating an addiction team into the management of patients transplanted for alcohol-associated liver disease reduces the risk of severe relapse

Jules Daniel, Jérôme Dumortier, Arnaud Del Bello, Lucie Gamon, Nicolas Molinari, Stéphanie Faure, Magdalena Meszaros, José Ursic-Bedoya, Lucy Meunier, Clément Monet, Francis Navarro, Olivier Boillot, Georges-Philippe Pageaux, Hélène Donnadieu-Rigole

Supplementary data:

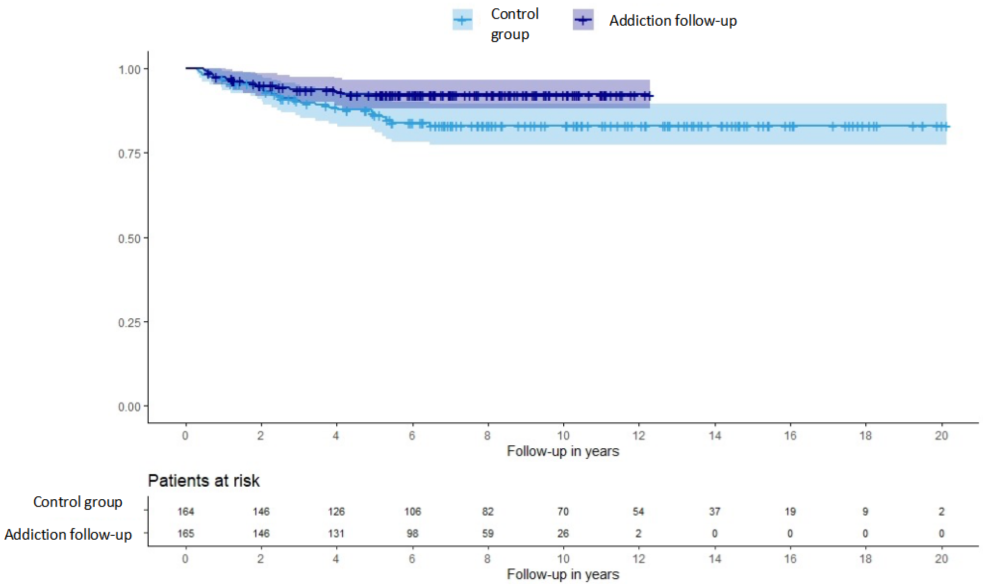

Supplement: Multimedia component 1 [file mmc1.pdf]
