# Supplementary material for: Integrating an addiction team into the management of patients transplanted for alcohol-associated liver disease reduces the risk of severe relapse
Source: JHEP Rep. 2023 Jul 30;5(10):100832. doi: 10.1016/j.jhepr.2023.100832 (PMC10480527; doi:10.1016/j.jhepr.2023.100832)
Supplement: Multimedia component 2 [file mmc2.zip › protocole J hep reports.docx]

Impact of integrating an addiction team on post liver transplantation survival for alcohol-associated liver disease and its complications: A multicenter retrospective comparative study

**NCT 04964687**

Liver transplantation (LT) is currently the only treatment available for advanced liver disease, and remains a first line treatment in hepatocellular carcinoma.

Among chronic liver diseases, alcohol-associated liver diseases, whether or not complicated by hepatocellular carcinoma, represent the leading cause of liver transplantation in Europe. It has been shown that the relapse of excessive alcohol consumption after LT, which affects 10 to 26% of patients, greatly affects the prognosis in terms of overall survival, via the more frequent occurrence of de novo cancers, cardiovascular events, recurrence of cirrhosis.

The integration of addiction physician in liver transplant units is far from being systematic in France.

Risk factors for relapsing alcohol consumption after liver transplantation have been identified: duration of pre-transplant abstinence, social isolation, lack of employment, psychiatric comorbidities, dependence on several products besides alcohol, non-compliance, young age. Using these risk factors, it is possible to identify patients with higher risk of relapse.

**Hypothesis:**

Integrating an addiction team into a LT unit improves the prognosis of patients with alcohol-related liver disease (ARLD) requiring LT. Our hypothesis is that patients managed by an addiction team before and after LT have less frequent alcohol relapses, thus decreasing the risk of cardiovascular complications, de novo cancer, recurrence of alcohol-related cirrhosis, and consequently increasing their overall survival.

**Objectives:**

- *Main objective:*

Identify the impact of the integration of an addiction team within liver transplant units on the overall survival of liver recipients with ALD as an indication.

- *Secondary objectives:*

Evaluate post-liver transplantation incidence of (i) alcohol relapse, (ii) sustained alcohol relapse, (iii) recurrence of alcohol-related cirrhosis, (iv) onset cardiovascular risk factors (hypertension, dyslipidemia, diabetes, smoking), (v) the occurrence of cardiovascular events (acute coronary syndrome, stroke, arteriopathy), (vi) and assess survival without the occurrence of *de novo* cancer.

**Outcomes**:

- *Primary outcomes:* Overall survival of patients transplanted for ARLD
- *Secondary outcomes*:
  - Alcohol relapse rate: Number of patients with alcohol relapse among all included patients
  - Sustained alcohol relapse rates: Number of patients with sustained alcohol relapse among all included patients
  - Rate of alcohol-related cirrhosis recurrence: Number of patients with alcohol-related cirrhosis recurrence among all included patients
  - Rate of development of cardiovascular diseases: Number of patients with cardiovascular disease among all included patients
  - Rate of de novo-cancer: Number of de novo cancer (associated or not with alcohol or tobacco consumption) among all included patients

**Methods:** This study was a retrospective comparison between centers with or without addiction monitoring. (Retrospective Cohort)

- We plan to compare two cohorts of liver transplant recipients, according to whether they received or not a specific addiction care before and after LT. These patients were identified from 3 centers: Montpellier University Hospital, Lyon University Hospital, Toulouse University Hospital

**Study population**:

*Inclusion criteria:*

- age >18 years,
- having received a LT between January 2000 and December 2015,
- ARLD for primary indication for LT or hepatocellular carcinoma (HCC) as primary indication for LT with ARLD as secondary indication,
- having survived for over 6 months after hospital discharge.

*Exclusion criteria:*

- association of ARLD with other causes of liver disease (such as chronic hepatitis B or C, hereditary hemochromatosis, auto-immune hepatitis, primary sclerosing cholangitis, primary biliary cholangitis, Caroli’s syndrome, Alpha-1 antitrypsin deficiency),
- death before hospital discharge after LT,
- Patients unwilling to participate to the study

**Data collected (anonymously):**

### *Pre-LT Data*

- Sociodemographic information: gender, marital status, children, professional status and distance of the patient residence from the LT unit
- Clinical data collected: Child-Pugh and Model for End-Stage Liver Disease (MELD) scores, history of HCC, hypertension, diabetes, dyslipidemia, and cardiovascular events
- Addiction data: Duration of alcohol abstinence before LT, Tobacco consumption

### *Post-LT data*

- Alcohol consumption after LT and classification of alcohol relapse (1) Sustained relapse: alcohol intake exceeding three portions per day for males and two for females, sustained for at least 100 days with a sense of loss of control, (2) non-sustained relapse: when alcohol intake was limited to small amounts (*ie*, <5 units per drinking occasion) with sobriety quickly recovered. We defined this pattern as “slips”. We reported this pattern as regular relapse when alcohol consumption was frequent and daily, for at least 100 days, but not excessive (*ie*, no more than 21 units a week for males, 14 for females).
- Tobacco consumption
- Medical data: initial then subsequent immunosuppressive regimen, history of graft rejection, the development of cardiovascular risk factors including hypertension, diabetes, dyslipidemia, and cardiovascular events (acute coronary syndrome, occlusive arterial disease, stroke). The development of *de novo* malignancy of any type after LT, *de novo* alcohol and/or tobacco-related malignancy (lung, otorhinolaryngologic, colorectal, anal, pancreatic, bladder, esophageal), *de novo* alcohol-related malignancy (otorhinolaryngologic and esophageal squamous cell carcinomas), or HCC recurrence after LT.

*Others:*

Date of death.

Date of latest news.

**Origin of the data collected:**

Paper and computerized medical records from participating centers. Bio Medicine Agency Cristal Database (French national agency dealing with the organ procurement and transplantation).

Circuit of collected data:

Collection of anonymized data by the project leader, shared with the senior study manager.

This anonymized data will then be shared with the DIM for statistical analysis.

Terms and duration of data retention as well as the collection medium and the terms of anonymization.

Data collected and stored on computer media (Excel file). Anonymized data: patients referenced in the format XXX – 000 with anonymization directory on a separate file).

The database will be kept on the CHU server for a period of 2 years after the publication of the results.

**Study plan**:

2 groups:

- The group of interest (liver transplant recipients seen by addiction team before and/or after LT) was composed of patients transplanted in Montpellier University Hospital since 2008, *i.e* since the integration of an addiction team in the LT unit.

The comparison group was composed of patients managed by LT teams that did not include an addiction specialist. These patients were identified from Montpellier center (2000-2007), Lyon center (2000-2010), Toulouse center (2008-2015).

Due to the retrospective, observational nature of the study and to minimize differences between groups, propensity score matching (PSM) analyzes was performed.
