# Supplementary material for: Integrating an addiction team into the management of patients transplanted for alcohol-associated liver disease reduces the risk of severe relapse
Source: JHEP Rep. 2023 Jul 30;5(10):100832. doi: 10.1016/j.jhepr.2023.100832 (PMC10480527; doi:10.1016/j.jhepr.2023.100832)
Supplement: Multimedia component 2 [file mmc2.zip › SAP.docx]

Statistical Analysis Plan

# Administrative information

# Impact of Integrating an Addiction Team on Post Liver Transplantation Survival for Alcohol-associated Liver Disease and Its Complications

**Sponsor**

CHU de Montpellier

Centre Administratif André Bénech

191, avenue du Doyen Gaston Giraud

34295 Montpellier cedex 5

**N° IDRCB :** IRB ID 202100883

**ClinicalTrials.gov Identifier:** NCT04964687

SAP version 1.0 (January 12^th^ 2023)

Protocol version 1.0

SAP revisions:

| Date | Version | Justification |
| --- | --- | --- |
| January 12^th^ 2023 | 1.0 | Creation date |
|  |  |  |

Roles and responsibility:

| Name | Role | Date | Signature |
| --- | --- | --- | --- |
| Nicolas Molinari | Person writing the SAP, senior statistician | January 12^th^ 2023 | 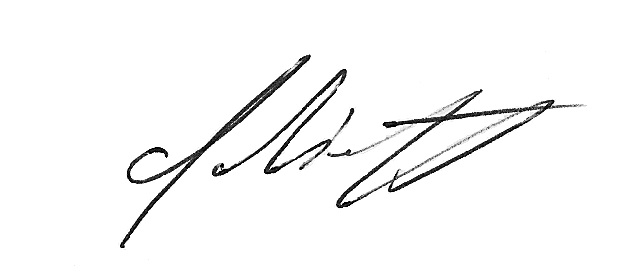 |
| Donnadieu Rigole Hélène | Chief investigator |  | 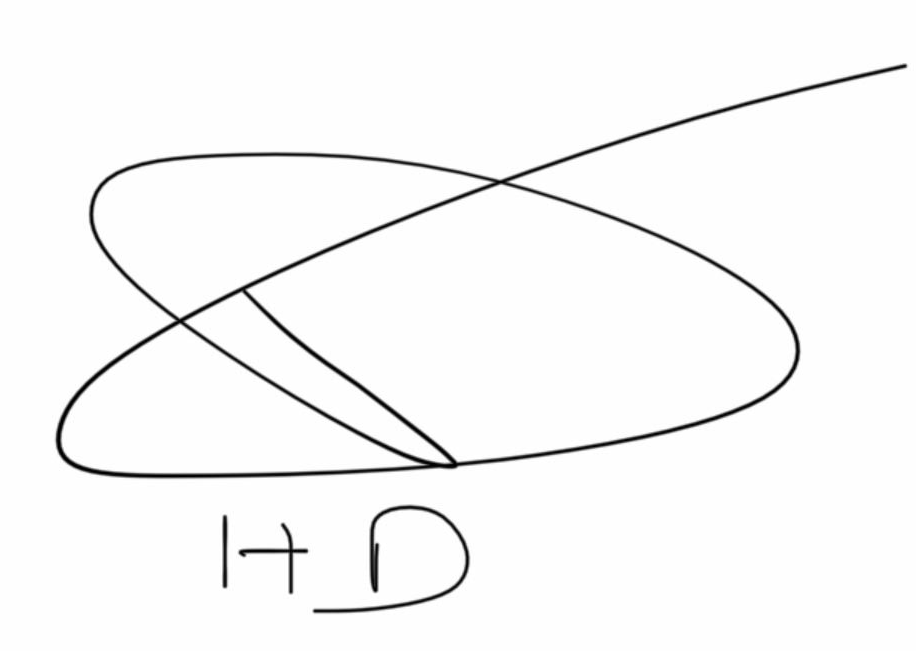 |

# Introduction

## Background and rationale

Liver transplantation is currently the only treatment available for advanced liver disease, and remains a first line treatment in hepatocellular carcinoma.

Among chronic liver diseases, alcohol-related liver diseases, whether or not complicated by hepatocellular carcinoma, represent the leading cause of liver transplantation in Europe. It has been shown that the resumption of excessive alcohol consumption after liver transplantation, which affects 10 to 26% of patients, greatly affects the prognosis in terms of overall survival, via the more frequent occurrence of de novo cancers, cardiovascular events, recurrence of cirrhosis.

The integration of addiction physician in liver transplant units is far from being systematic in France.

Risk factors for resuming alcohol consumption after liver transplantation have been identified: duration of pre-transplant abstinence, social isolation, lack of employment, psychiatric comorbidities, dependence on several products, non-compliance, young age. They make it possible to identify patients most at risk of relapse.

Integrating an addiction team into a liver transplantation (LT) unit improves the prognosis of patients with alcohol-related liver disease (ARLD) requiring liver transplantation. Our hypothesis is that patients managed by an addiction team before and after liver transplantation have less frequent alcohol relapses, thus decreasing the risk of cardiovascular complications, de novo cancer, recurrence or alcohol-related cirrhosis, and consequently increasing their overall survival.

## Objectives

- *Main objective:*

Identify the impact of the integration of an addiction team within liver transplant units on the overall survival of liver transplant patients with alcohol-associated liver disease as an indication.

- *Secondary objectives:*

Evaluate post-liver transplantation the incidence of (i) alcohol relapse, (ii) sustained alcohol relapse, (iii) recurrence of alcohol-related cirrhosis, (iv) onset cardiovascular risk factors (hypertension, dyslipidemia, diabetes, smoking), (v) the occurrence of cardiovascular events (coronary syndrome, stroke, arteriopathy), (vi) and assess survival without the occurrence of cancer of new

# Study Methods

## Trial design

In this observational, retrospective and multicentre study, investigator seek to determine the effect of integrating an addiction team into liver transplantation unit on prognosis of patients with alcohol-related liver disease requiring liver transplantation. Investigator plan to compare patients in 2 groups, depending on whether they have received or not specific addiction care before and after transplantation. This study was conducted over a period of 15 years in three French liver transplant units.

## Randomization

NA

## Sample size

A total of 1840 patients received a LT over the study periods defined for each center. Among these, 611 patients (whole cohort) were enrolled in the study. Among these 611 patients, 190 were managed by the addiction team.

# Statistical Principals

## Adherence and Population deviations

Enrolled in a program with follow-up *vs*. non-follow-up

## Definition of adherence to the intervention and how this is assessed including extent

The pre-transplant addictology consultation was mandatory and was part of the pre-transplant check-up. If the consultation was not carried out, it was rescheduled.

## Analysis populations

Statistical analysis will be performed in an intention to-treat population including all the included patients except patients who withdraw their consent or do not meet the inclusion criteria.

Matched cohorts will be defined to perform a quasi-experiment study. Propensity score matching (PSM) analyzes will be performed in a 1:1 ratio for the following patient characteristics: sex, age (+/-56 years), smoking (non-smokers/active or former smokers), history of HCC (yes/no), duration of alcohol abstinence before LT (+/-6 months), cardiovascular risk factors (yes/no), MELD score (+/-18), and Child-Pugh score.

# Trial Population

## Eligibility criteria

An individual must fulfill all of the following criteria at the time of study enrollment in order to be eligible:

- age >18 years,
- having received a LT between January 2000 and December 2015,
- ARLD for primary indication for LT or hepatocellular carcinoma (HCC) as primary indication for LT with ARLD as secondary indication,
- having survived for over 6 months after hospital discharge.

Patients fulfilling one or more of the following criteria will not be included

- association of ARLD with other causes of liver disease (such as chronic hepatitis B or C, hereditary hemochromatosis, auto-immune hepatitis, primary sclerosing cholangitis, primary biliary cholangitis, Caroli’s syndrome, Alpha-1 antitrypsin deficiency),
- death before hospital discharge after LT,
- Patients unwilling to participate to the study

The flow diagram will be presented according to the following figure.


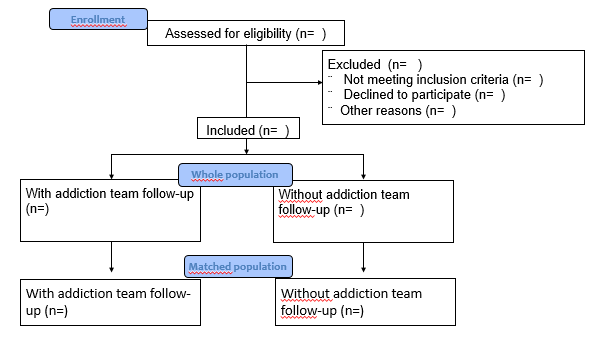


Baseline patient characteristics: socio-demographic data will be collected upon enrolment.

# Analysis

## Outcome definitions

The primary outcome is the all-cause mortality.

The secondary outcomes will be

- Alcohol relapse rate: Number of patients with alcohol relapse among all included patients
- Sustained alcohol relapse rates: Number of patients with sustained alcohol relapse among all included patients
- Rate of alcohol-related cirrhosis recurrence: Number of patients with alcohol-related cirrhosis recurrence among all included patients
- Rate of development of cardiovascular diseases: Number of patients with cardiovascular disease among all included patients
- Rate of de novo-cancer: Number of de novo cancer (associated or not with alcohol or tobacco consumption) among all included patients

## Analysis methods

All analyses will be conducted by the medical statistical department of the Montpellier University Hospital using statistical software (SAS Enterprise Guide, version 7.13, or statistical software R, version 4.0.3). A two-sided p value of less than 0.05 will be considered to indicate statistical significance.

**Description of the patient groups at baseline:** the baseline features of the overall population and of each group will be described, using n (%) for categorical variables and the minimum, maximum, mean, SD, median and quartiles for quantitative variables.

**Matching :** the GREEDY NEAREST NEIGHBOR 1:1 with « caliper » of 0.2 will be used to match exposed to non exposed patients.

**Primary Analysis:** The survival time will be described by the Kaplan-Meier method and compared with a log-rank test. A Cox proportional-hazards model will be used to calculate hazard ratios (HRs) for death. Cox regression models will be adjusted on relevant baseline covariates. Covariates will be selected in a backward selection procedure if p<0⋅15 in the univariate analysis and then presented as adjusted HRs with 95% Confidence Intervals (CIs).

**Secondary Analyses:** we will perform unadjusted analyses with regard to each of the pre-specified secondary and exploratory outcomes. Continuous outcomes will be compared with the Mann-Whitney rank-sum test and categorical variables with the chi-square test. Logistic and Cox regression models will be adjusted on relevant baseline covariates. Covariates will be defined as binary variables and continuous variables dichotomized according to their median tested in the model, and will be selected in a backward selection procedure if p<0⋅15 in the univariate analysis and then presented as adjusted ORs or HRs with 95% Confidence Intervals (CIs).

**Missing Data:** based on the prior trial performed in similar settings, we anticipate less than 5% missing data for the primary outcome. Missing data will not be imputed. Analyses will be performed on the complete cases. We will indicate in each table the number of observed data.

# Data sharing

Research data and other material (eg, study protocol and statistical analysis plan) will be made available to the scientific community, immediately on publication, with as few restrictions as possible. All requests should be submitted to the corresponding author who will review with the other investigators for consideration. A data use agreement will be required before the release of participant data and institutional review board approval as appropriate.
