# Supplementary material for: Integrating an addiction team into the management of patients transplanted for alcohol-associated liver disease reduces the risk of severe relapse
Source: JHEP Rep. 2023 Jul 30;5(10):100832. doi: 10.1016/j.jhepr.2023.100832 (PMC10480527; doi:10.1016/j.jhepr.2023.100832)

Research article

JHEP|Reports

Integrating an addiction team into the management of patients transplanted for alcohol-associated liver disease reduces the risk of severe relapse

Jules Daniel,[1] Jérôme Dumortier,[2] Arnaud Del Bello,[3] Lucie Gamon,[4] Nicolas Molinari,[4,5] Stéphanie Faure,[1] Magdalena Meszaros,[1] José Ursic-Bedoya,[1] Lucy Meunier,[1] Clément Monet,[6] Francis Navarro,[5,7] Olivier Boillot,[2] Georges-Philippe Pageaux,[1,5] Hélène Donnadieu-Rigole[5,8,*]

[1]Hepatology and Liver Transplantation Department, Saint Eloi Hospital, University Hospital of Montpellier, Montpellier, France; [2]Fédération des Spécialités Digestives, Hôpital Edouard Herriot, Hospices Civils de Lyon et Université Claude Bernard, Lyon, France; [3]Nephrology and Organ Transplant Department, CHU de Toulouse, Toulouse, France; [4]Medical Information Department, La Colombière Hospital, University Hospital of Montpellier, Montpellier, France; [5]Medical University of Montpellier (UM1), Montpellier, France; [6]Department of Anesthesia and Intensive Care unit, University Hospital of Montpellier, St-Eloi Hospital, University of Montpellier, PhyMedExp, INSERM U1046, CNRS UMR, Montpellier, France; [7]Department of Digestive Surgery, University Hospital of Montpellier, St-Eloi Hospital, Montpellier, France; [8]Addictions Department, Saint Eloi Hospital, University Hospital of Montpellier, Montpellier, France

JHEP Reports **2023.** https://doi.org/10.1016/j.jhepr.2023.100832

Background & Aims: Liver transplantation (LT) is a last resort treatment for patients at high risk of mortality from end-stage liver disease. Over the past years, alcohol-associated liver disease has become the most frequent indication for LT in the world. The outcomes of LT for alcohol-associated liver disease are good, but return to alcohol use is detrimental for medium-term survival because of cancer development, cardiovascular events, and recurrent alcohol-associated cirrhosis. Several strategies have been developed to prevent return to alcohol use during the pre- or post-LT period, but there are no specific recommendations. Therefore, the main objective of this study was to investigate if the integration of an addiction team in a LT unit affected the rate of severe alcohol relapse after LT. The secondary objectives were to assess the effects of addiction follow up on cardiovascular events, cancer, and overall survival.
Methods: This study was a retrospective comparison between centres with or without addiction monitoring.
Results: The study included 611 patients of which 79.4% were male with a mean age of 55.4 years at the time of LT, 190 were managed by an integrated addiction team. The overall alcohol relapse rate was 28.9% and the rate of severe relapse was 13.0%. Patients with addiction follow-up had significantly less frequent severe alcohol relapse than those in the control group (p = 0.0218). Addiction follow up (odds ratio = 0.19; p = 0.001) and age at LT (odds ratio = 1.23; p = 0.02) remained significantly associated with post-LT cardiovascular events.
Conclusions: Our study confirms the benefits of integrating an addiction team to reduce return to alcohol use after LT.
Clinical Trials registration: This study is registered at ClinicalTrials.gov (NCT 04964687).
Impact and implications: The main indication for liver transplantation is alcohol-associated cirrhosis. There are currently no specific recommendations on the addiction monitoring of transplant candidates, although severe return to alcohol use after liver transplantation has a negative impact on long-term survival of patients. In this study, we explored the impact of a systematic addiction intervention on the return to alcohol use rates. In our transplantation centre, we demonstrated the interest of an addiction follow up to limit the severe alcohol relapses rate. This information should be further investigated in prospective studies to validate these data.
© 2023 The Author(s). Published by Elsevier B.V. on behalf of European Association for the Study of the Liver (EASL). This is an open access article under the CC BY license (http://creativecommons.org/licenses/by/4.0/).

UNCORRECTED PROOF

Introduction

Liver transplantation (LT) is a last resort treatment for patients at high risk of death from end-stage liver disease (ESLD). Nonetheless, LT currently remains the most efficient treatment and frequently the only available treatment. Over the past years, alcohol-associated liver disease (ALD) has become the most frequent indication for LT in Europe, including France, and in the United States.[1,2] The outcomes of LT for ALD are good, but a

Keywords: Alcohol relapse; Liver transplantation; Addiction monitoring; Survival.
Received 14 April 2023; received in revised form 24 May 2023; accepted 14 June 2023; available online XXX
* Corresponding author. Address: Addictions Department, Saint Eloi Hospital, University Hospital of Montpellier, 80 rue Augustin Fliche, 34295 Montpellier CEDEX 5, France. Tel.: +33-0467337020; fax: +33-0467337869.
E-mail address: h-donnadieu@chu-montpellier.fr (H. Donnadieu-Rigole).

ELSEVIER

EASL The Home of Hepatology

severe alcohol relapse has a significant effect on survival beyond 5 years after LT.[3,4] The reported rates of alcohol relapse after LT vary between studies because of the different definitions[5] used to classify drinking patterns: ‘any use’, ‘slips’, or ‘relapse’ which can be ‘severe’ or ‘regular’ depending on the case.[4] Studies suggest return to alcohol use can reach up to 40% of patients with ALD in the 5 years following LT.[6] Only severe alcohol relapse, with an estimated frequency between 11% and 26%, has a negative effect on long-term survival after LT. This is irrespective of the primary indication of LT and concerns return to alcohol use occurring both early in the first months after LT and later in the years following surgery.[4,7,8] Among recipients with ALD, severe relapse after LT leads to impaired long-term survival through recurrent alcohol-associated cirrhosis (RAC), cardiovascular events, and *de novo* solid-organ malignancies.[4,9–11]

Several strategies have been developed to prevent return to alcohol use. During the pre-LT period, many well-described risk factors allow for identification of a subgroup of vulnerable patients: social determinants (*e.g.* lack of social stability, unemployment, loneliness), male sex, psychiatric comorbidities, polysubstance abuse, duration of alcohol abstinence before LT, non-compliance with medical care, and young age.[8,12–15] After LT, integrating an addiction team in the LT programme has been advocated by the latest guidelines in Europe and the United States.[16–19] The aim is to manage alcohol-use disorder (AUD) within transplantation units via combining psychosocial and pharmacological interventions as previously reported by the American Association for the Study of Liver Diseases guidelines.[20,21]

The main objective of this study was therefore to describe whether the integration of an addiction team in a LT unit affected the rate of severe alcohol relapse after LT. The secondary objectives were to assess the risk factors associated with severe relapse and the effects of addiction follow up on cardiovascular events, cancer, and survival.

Patients and methods

The study protocol was approved by the Institutional Review Board of Montpellier University Hospital (IRB ID 202100883). This IRB is available in France for all centres implicated in this study (Loi jardé). The study was carried out in accordance with the Declaration of Helsinki revised in 2008.

Study cohort

Data collection was conducted from October 2019 to May 2021 in the LT units of Montpellier, Lyon, and Toulouse University Hospitals (France). These units perform between 40 and 100 LTs each year. Data were obtained from electronic medical records available at each unit.

The inclusion criteria were: age >18 years, having received a LT between January 2000 and December 2015, ALD for primary indication for LT or hepatocellular carcinoma (HCC) as primary indication for LT with ALD as secondary indication, and having survived for over 6 months after hospital discharge. The exclusion criteria were: association of ALD with other causes of liver disease (such as chronic hepatitis B or C, hereditary hemochromatosis, auto-immune hepatitis, primary sclerosing cholangitis, primary biliary cholangitis, Caroli’s syndrome, Alpha-1 antitrypsin deficiency), death before hospital discharge after LT.

Because of the different addiction management strategies that have evolved over time, two cohorts were studied and compared. The group of interest was composed of patients who underwent transplantation in Montpellier University Hospital since 2008, that is, since the integration of an addiction team in the LT unit. The follow up after LT was performed by the same addiction specialist and LT team clinicians. The addictions department has a codified LT patient pathway with priority addiction appointments and joint meetings that commence before LT. Almost all patients consulted with the addiction team; some patients could not be evaluated before LT as a result of severe symptoms (intensive care) or symptoms of hepatic encephalopathy. These patients were all subsequently given the opportunity to seek specific management and thus remained included in the group of interest. The comparison group was composed of patients managed by LT teams that did not include an addiction specialist. These patients were identified from several centres: (1) the Montpellier centre (2000–2007), that is, before the integration of an addiction team in the LT unit, (2) the Edouard Herriot University Hospital in Lyon (2000–2010), and (3) the Rangueil University Hospital in Toulouse (2008–2015). Both the Lyon and Toulouse centres did not have systematic addictology follow up but offered addiction consultation on request in the case of return to alcohol use.

Data collected

Pre-LT data

Pre-LT data were collected for all patients included. Sociodemographic information included sex, marital status (single or in a relationship), whether or not the patient had children, professional status (employed, unemployed [described as inactive] or retired [described as inactive]), and distance of the patient’s residence from the LT unit according to the administrative divisions of France named departments (same department, bordering department, or distant department).

Clinical data collected were Child–Pugh and model for end-stage liver disease (MELD) scores, history of: HCC, hypertension, diabetes, dyslipidaemia, and cardiovascular events (including acute coronary syndrome, occlusive arterial disease, stroke).

For addiction data, the duration of alcohol abstinence before LT was divided into two categories: abstinence <6 or ≥6 months. Tobacco consumption was noted. A distinction was made between ‘active smokers’ (smoking ≥one cigarette per day in the month preceding LT) ‘former smokers’ (no longer smoking at the time of the interview but had smoked >100 cigarettes in their lifetime), and ‘non-smokers’ (having smoked <100 cigarettes in their lifetime). A distinction was then made between ‘non-smokers’ and ‘active or former smokers’. Total tobacco consumption was reported in terms of pack-years, calculated by multiplying the number of packs of cigarettes smoked per day by the number of years the person had smoked.

Post-LT data

All patients received follow up by the LT team in the first few years after LT. The delays between follow ups became longer as the time period from the date of LT increased. In the group of interest, follow up was supplemented by an addiction follow up during the pre- and post-LT period according to patient requirements. During the follow up consultations, the patients were advised to maintain complete abstinence from alcohol and were also offered assistance to stop smoking if applicable.

Patient alcohol consumption after LT was investigated using reports from follow-up visits with the LT team, during which

UNCORRECTED PROOF

FLA 5.6.0 DTD ■ JHEPR100832_proof ■ 12 August 2023 ■ 6:28 pm ■ ce

alcohol consumption was assessed by patient responses during oral interviews and statements provided by the patient relatives. No blood or urine tests were performed. In cases of return to alcohol use, alcohol intake was quantified by patient self-reported standard-units per day (1 unit = 10 g of alcohol) over the course of time. Among the different patterns described for alcohol consumption after LT, we distinguished several returns to alcohol use:[4]

(1) Severe relapse: alcohol intake exceeding three units per day for males and two for females, sustained for at least 100 days with a sense of loss of control.
(2) Non-severe relapse: when alcohol intake was limited to small amounts (*i.e.* <5 units per drinking occasion) with sobriety quickly recovered. We defined this pattern as 'slips'. We reported this pattern as regular relapse when alcohol consumption was frequent, for at least 100 days, but not excessive (*i.e.* no more than 21 units a week for males, 14 for females).

The medical data collected were initial then subsequent immunosuppressive regimen, history of graft rejection, the development of cardiovascular risk factors including hypertension, diabetes, dyslipidaemia, and cardiovascular events (acute coronary syndrome, occlusive arterial disease, stroke). We also noted the development of *de novo* malignancy of any type after LT, *de novo* alcohol and/or tobacco-related malignancy (lung, otorhinolaryngologic, colorectal, anal, pancreatic, bladder, oesophageal), *de novo* alcohol-related malignancy (otorhinolaryngologic and oesophageal squamous cell carcinomas), or HCC recurrence after LT.

Intervention in the group of interest

The addiction follow up proposed in the case of LT was a multidisciplinary approach to prevent return to alcohol use as previously recommended:[22] behavioural therapy, including cognitive behavioural therapy and motivational enhancement therapy. During the pre-LT period, all transplant candidates were Q5 seen by the addiction specialist. The BRENDA approach was used.[23] This type of interview makes it possible to carry out a psychosocial evaluation, a report of findings from the evaluation given to the patient, to address patient's needs, to assess patient reaction to advice and adjust the treatment plan as needed. The interview took place in a warm, empathetic, and non-judgmental environment. Open-ended questions, reformulation, and summaries were used. The following information was retrieved during the first interview in the pre-LT period: history of addictive or problematic behaviours, first degree history of addictive disease, age of first alcohol use, number of alcohol withdrawals, age of perception of problematic use, duration of problematic use and or use disorder before abstinence, addictive comorbidities (tobacco, other psychoactive substances, and behavioural addictions), duration of pre-transplant abstinence >6 months or <6 months, commitment to maintain abstinence from alcohol and belief in their ability to do so, psychological comorbidities, personal and interpersonal resources, presence of familial or social support considered satisfactory by the participant (declarative), professional activity. The following risk factors to return to alcohol use were collected as well as the personal resources: social determinants (lack of social stability, unemployment, or loneliness), male gender, psychiatric comorbidities, polysubstance abuse, duration of alcohol abstinence before LT (>6 months or <6 months), non-compliance with medical care, young age. After this first consultation, if the addictologist noted the presence of more than two risk factors for return to alcohol use, close follow up was proposed to the participant. The choice of this number of risk factors was made on the basis of clinical experience in the addictological follow up of transplant patients for over 20 years. This follow up included all the resources of addictology care; outpatient consultations with motivational interview at least every 4 weeks before and after LT, and use of addictolytic drugs if necessary[24] (camprosate: reduces the level of dependence and neuroprotection, limits cravings and their intensity; naltrexone: anti-craving effect; baclofen: anti-craving effect). In the case of a return to alcohol use or a period of high vulnerability, specific hospitalisations could be scheduled. Tobacco use was be systematically addressed and all measures to help cessation could be undertaken during follow up (telephone and dietician reminders, discussion groups) and nicotine-replacement drugs were also offered. Psychoactive substance use was discussed and support in changing consumption was offered using comprehensive therapies (speci- Q6 alised care centre, opiate substitution treatment, hospital withdrawal).

Statistical analysis

Pre-LT data are reported as frequencies (%), mean ± standard deviation, or medians (IQR 25–75%) as appropriate. Comparisons between addiction follow-up and control groups were performed using the Chi-squared test for categorical data or the Mann–Whitney U test for non-parametric data.

Because of the retrospective, observational nature of the study and to minimise differences between groups, propensity score matching (PSM) analyses were performed in a 1:1 ratio for the following patient characteristics: sex, age (±56 years), smoking (non-smokers/active or former smokers), history of HCC (yes/no), duration of alcohol abstinence before LT (±6 months), cardiovascular risk factors (yes/no), MELD score (±18), and Child–Pugh score. Validation of the matched pairs was confirmed by comparing variables using the McNemar's or Wilcoxon tests as appropriate.

The following analyses were performed on the whole cohort and on the matched cohort: severe alcohol relapse, overall survival, and cancer development were assessed by survival analyses performed according to the Kaplan–Meier method and compared using the log-rank test. The hazard ratio (HR) of receiving addiction team support was obtained using Cox regression models. Logistic regression was used to assess the effects of integrating a post-LT addiction team on the risk of cardiovascular events and to identify different risk factors for cardiovascular events. A multivariate model was established. Variables were selected if the p value was <0.15 in the univariate analysis and a backward selection procedure was used to select the final model (coherence between backward and stepwise analysis for multivariate analyses of severe alcohol relapse was made). A value of p ≤0.05 for all other analyses was considered statistically significant. Statistical analyses were conducted using SAS software version 9.1 (SAS Institute, Cary, NC, USA).

Results

A total of 1,840 patients received an LT over the study periods defined for each centre. Among these, 611 patients (whole cohort) were enrolled in the study. Among these 611 patients,

UNCORRECTED PROOF

FLA 5.6.0 DTD ■ JHEPR100832_proof ■ 12 August 2023 ■ 6:28 pm ■ ce

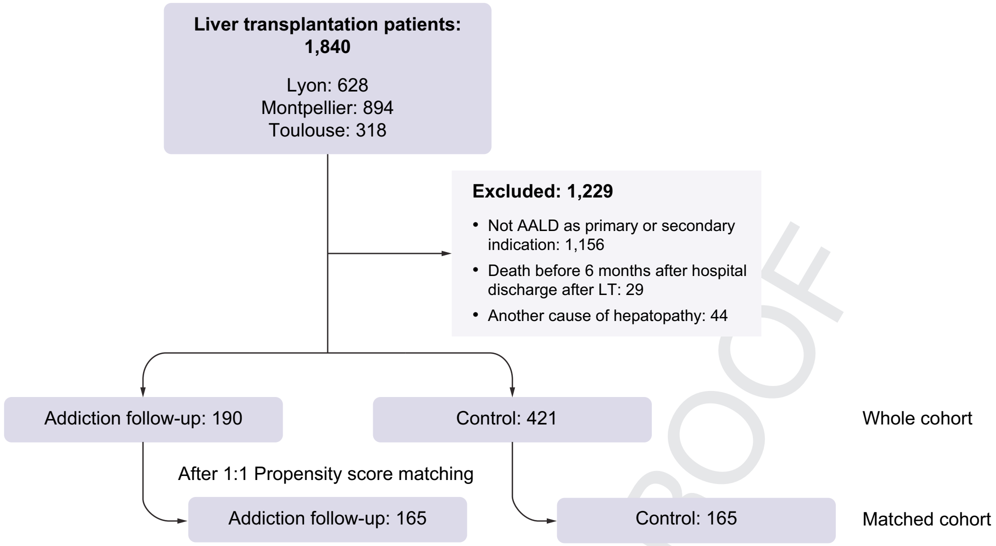

web 4C/FPO

Fig. 1. Study flow chart. Q16

190 were managed by the addiction team. Patient exclusions were for the following: ALD was not the primary or secondary indication for LT (n = 1,156), death within 6 months or before hospital discharge (n = 29), and association of ALD with another causes of liver disease (n = 44). After PSM analysis, 165 patients with addiction follow up and 165 control patients without any addiction management were compared (matched cohort) (Fig. 1).

Pre-LT features

The patients in the whole cohort were in majority male (79.4%) with a mean age at the time of LT of 55.4 years (Table 1). Most of the transplanted patients (58.2%) had a Child–Pugh C score at the time of LT. The mean value of the MELD score was 20. HCC was detected in 32.9% of patients and 8.1% had a history of severe cardiovascular events. The duration of alcohol abstinence before LT was over 6 months for 85.4% of patients. Most patients (76.3%) Q7 had a prior history of smoking with an average of 23.4 pack-years and 37.9% still smoked shortly before LT (Table 2).

In the addiction follow-up group, 84% of patients (160/190) were seen before LT by the addiction team. In certain situations, linked to liver disease severity (severe alcohol associated hepatitis not responding to steroids and/or intensive care), the transplant candidate could not be evaluated by the addiction team before LT. After LT, 68% of patients were followed in addictology in addition to their classic follow up by the hepatologist.

Comparing the control group to the addiction follow-up group before PSM analysis, patients in the addiction follow-up group at the time of the LT were younger, more likely to have a more advanced liver disease according to Child–Pugh and MELD scores, to have HCC, to have a history of smoking, and have a shorter duration of alcohol abstinence before LT. After PSM analysis, both groups were comparable for all aforementioned variables tested by calculation of propensity scores.

Post-LT features

Return to alcohol use rates and risk factors

In the whole cohort, the overall return to alcohol use rate (severe and non-severe) was 28.9% (182/611) and the rate of severe relapse was 13.0% (79/611). Patients with addiction follow-up had significantly less frequent severe alcohol relapse than those in the control group (6.8% *vs.* 15.8%, HR 0.50 [0.27–0.90]; p = 0.0218). The median time to onset of return to alcohol use was 8.1 years (9.5 for the control group and 6.5 for the addiction follow-up group). Kaplan–Meier analyses are represented in

Table 1. Baseline characteristics of the addiction follow-up group and control group patients before and after propensity score matching analysis.

Variables	Whole cohort			Matched cohort		
	Addiction follow-up group (n = 190)	Control group (n = 421)	*p* value	Addiction follow-up group (n = 165)	Control group (n = 165)	*p* value
Age, mean (range)	56.2 (±7.9)	55.0 (±7.1)	0.03	55.8 (±8.2)	56.1 (±6.5)	0.61
Male sex	154 (81.1%)	331 (78.6%)	0.49	133 (80.6%)	134 (81.2%)	0.76
Married/in a relationship	130 (68.4%)	257 (61.0%)	0.59	118 (71.5%)	101 (61.2%)	0.52
With children	153 (80.5%)	325 (77.2%)	0.47	137 (83.0%)	130 (78.8%)	0.13
Employed	37 (19.5%)	72 (17.1%)	0.73	35 (21.2%)	34 (20.6%)	0.77
Department of residence			0.69			0.50
Same	88 (46.3%)	187 (44.4%)		79 (47.9%)	69 (41.8%)	
Bordering	68 (35.8%)	146 (34.7%)		57 (34.5%)	59 (35.8%)	
Distant	34 (17.9%)	88 (20.9%)		29 (17.6%)	37 (22.4%)	

Chi-squared test for categorical data, Wilcoxon–Mann–Whitney test for continuous data.

Table 2. Medical and addiction data at the time of liver transplantation (LT) for the addiction follow-up group and control patient group before and after propensity score matching.

Variables	Whole cohort		*p* value	Matched cohort		*p* value
	Addiction follow-up group (n = 190)	Control group (n = 421)		Addiction follow-up group (n = 165)	Control group (n = 165)	
Child–Pugh score			<0.01			0.39
A	24 (12.6%)	57 (13.5%)		24 (14.5%)	24 (14.5%)	
B	37 (19.5%)	137 (32.5%)		37 (22.4%)	35 (21.2%)	
C	129 (67.9%)	226 (53.7%)		104 (63.0%)	106 (64.2%)	
MELD score, mean (range)	21.3 (±8.8)	18.4 (±8.3)	<0.01	20.1 (±8.4)	20.2 (±9.0)	0.86
Hepatocellular carcinoma	78 (41.1%)	123 (29.2%)	<0.01	73 (44.2%)	78 (47.3%)	0.22
Abstinence >6 months before LT	135 (71.1%)	375 (89.1%)	<0.01	134 (81.2%)	136 (82.4%)	0.59
Active or former smoker	156 (82.1%)	305 (72.4%)	0.04	135 (81.8%)	128 (77.6%)	0.07
Hypertension	55 (28.9%)	109 (25.9%)	0.45	46 (27.9%)	56 (33.9%)	0.26
Diabetes	44 (23.2%)	98 (23.3%)	0.95	40 (24.2%)	49 (29.7%)	0.17
Dyslipidaemia	14 (7.4%)	33 (7.8%)	0.83	11 (6.7%)	16 (9.7%)	0.30
Previous cardiovascular events	15 (7.9%)	34 (8.1%°	0.93	14 (8.5%)	15 (9.1%)	0.85

Chi-squared test for categorical data and the Wilcoxon–Mann–Whitney test for continuous data.
LT, liver transplantation; MELD, model for end-stage liver disease.

Fig. 2. In the matched cohort, 15.2% for the control group and 7.3% for the addiction follow-up group had severe relapse, and similarly, addiction follow up had a lowering effect on rate of severe relapse (Fig. S1).

We studied the factors influencing severe relapse (found in Tables 3 and 4). Addiction follow up, being in a relationship, abstinence for >6 months, and older age at LT were significantly associated with a reduced risk of severe alcohol relapse in the whole cohort.

Cardiovascular events

A total of 145 cardiovascular events occurred in 119 patients (19.5%) after LT in the whole cohort (22.3% in the control group and 13.2% in the addiction follow-up group): 53 acute coronary syndrome events, 60 occlusive arterial disease events, and 33 stroke events.

In the whole cohort, severe post-LT cardiovascular events were significantly associated with addiction follow-up (p = 0.008), male sex (p = 0.033), HCC (p = 0.019), pre-LT period active

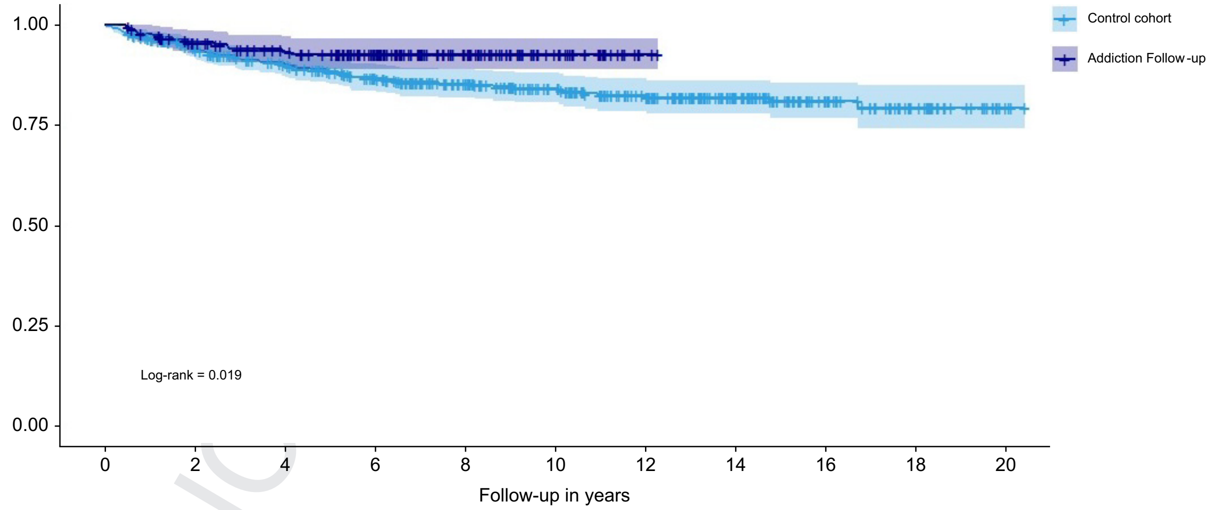

Patients at risk											
Control cohort	164	146	126	106	82	70	54	37	19	9	2
Addiction follow-up	165	146	131	98	59	26	2	0	0	0	0

Follow-up time, years	Control cohort		Addiction follow-up cohort	
	No relapse	Relapse	No relapse	Relapse
0	1	0	1	0
1	0.9634	0.0366	0.9757	0.0243
5	0.8625	0.1375	0.9229	0.0771
10	0.8416	0.1684	0.9229	0.0771

web 4C/FPO

Fig. 2. Kaplan–Meier analysis comparing the risk of severe alcohol relapse between the addiction follow up and control patient groups from within the whole cohort. *p* value (log-rank) = 0.019.

Table 3. Univariate (*p* <0.15) and multivariate (*p* <0.05) analyses of sustained alcohol relapse using logistic regression before and after propensity score matching analysis.

Variables (univariate)	Whole cohort			Matched cohort		
	HR	95% CI	*p* value	HR	95% CI	*p* value
Addiction follow-up	0.50	[0.27–0.90]	0.0218	0.47	[0.25–0.89]	0.0195
Age at LT	0.94	[0.92–0.97]	<0.0001	0.94	[0.91–0.96]	0.052
Male sex	1.10	[0.63–1.90]	0.745	1.11	[0.63–1.96]	0.726
Married/in a relationship	0.46	[0.29–0.72]	0.001	0.46	[0.29–0.73]	0.005
With children	0.89	[0.49–1.62]	0.706	0.83	[0.46–1.52]	0.549
Unemployed	1.50	[0.77–2.92]	0.233	1.49	[0.76–2.90]	0.243
Hepatocellular carcinoma (pre-LT)	0.67	[0.40–1.13]	0.135	0.63	[0.36–1.09]	0.099
Abstinence >6 months before LT	0.53	[0.31–0.89]	0.017	0.30	[0.14–0.62]	0.001
Active or former smoker before LT	1.20	[0.70–2.07]	0.503	1.24	[0.70–2.19]	0.459
Hypertension	0.56	[0.31–1.01]	0.055	0.49	[0.27–0.92]	0.026
Previous cardiovascular events	0.33	[0.08–1.35]	0.123	0.33	[0.08–1.33]	0.119
Smoking after LT	2.78	[1.77–4.36]	<0.0001	2.76	[1.75–4.34]	<0.0001
**Variables (multivariate)**	**HR**	**95% CI**	***p* value**	**HR**	**95% CI**	***p* value**
Addiction follow-up	0.42	[0.22–0.79]	0.007	0.41	[0.21–0.79]	0.008
Married/in a relationship	0.59	[0.37–0.94]	0.028	0.58	[0.36–0.93]	0.022
Abstinence >6 months before LT	0.46	[0.26–0.81]	0.007	NA	NA	NA
Age at LT	0.95	[0.93–0.98]	0.001	0.94	[0.92–0.97]	<0.0001

Variables were selected if the *p* value was <0.15 in the univariate analysis and a backward selection procedure was used to select the final model. A value of *p* ≤0.05 for all other analyses was considered statistically significant.
HR, hazard ratio; LT, liver transplantation.

Table 4. Coherence between backward and stepwise analysis for multivariate analyses of sustained alcohol relapse.

Variables		Multivariate (Backward)			Multivariate (Stepwise)		
		HR	95% CI	*p* value	HR	95% CI	*p* value
Addiction follow up	No	**1**		**0.0197**	**1**	**–**	**0.0314**
	Yes	**0.43**	**[0.22–0.88]**		**0.466**	**[0.233–0.934]**	
Married/in a relationship	Alone	1	–	0.0055	**1**	**–**	0.0021
	Couple	0.38	[0.19–0.75]		0.343	**[0.174–0.678]**	
Abstinence before LT	<6 months	1	–	0.0191	**1**	**–**	0.0023
	>6 months	0.43	[0.21–0.87]		0.348	**[0.177–0.686]**	
Age at LT		0.96	[0.91–1.00]	0.0529			

HR, their 95% CIs and the associated *p* value were calculated using Cox regression models.
HR, hazard ratio; LT, liver transplantation.

Table 5. Causes of death according to the different groups or whole cohort.

	Addiction follow-up group N = 190	Control group N = 421	Whole cohort
Recurrent alcohol cirrhosis	3/49 (6.1%)	10/187 (5.3%)	13/236 (5.5%)
Hepatocellular carcinoma	8/49 (16.3%)	12/187 (6.4%)	20/236 (8.5%)
Non-hepatic cancer	23/49 (46.9%)	90/187 (48.1%)	113/236 (47.9%)
Cardiovascular events	6/49 (12.2%)	10/187 (5.3%)	16/236 (6.8%)
Infection	4/49 (8.2%)	31/187 (16.6%)	35/236 (14.8%)
Graft rejection	0/49 (0.0%)	4/187 (2.1%)	4/236 (1.7%)
Other	5/49 (10.2%)	30/187 (16.0%)	35/236 (14.8%)
Total	49	187	236

or former smoking (*p* = 0.001), pre-LT period cardiovascular events (*p* <0.0001), hypertension (*p* = 0.001), diabetes (*p* = 0.0003), dyslipidaemia (*p* ≤0.0001), age at LT (*p* = 0.028), and MELD score >18. In multivariate analysis, severe post-LT cardiovascular events were significantly associated with addiction follow-up (*p* = 0.0018), active or former smoking at LT (*p* = 0.048), pre-LT dyslipidaemia (*p* = 0.012), pre-LT cardiovascular events (*p* <0.0001), and pre-LT diabetes (*p* = 0.013).

At least one event occurred among 57 patients (17.3%) in the matched cohort. In multivariate analysis, only addiction follow up (odds ratio [OR] = 0.19; *p* = 0.001) and age at LT (OR = 1.23; *p* = 0.02) remained significantly associated with post-LT cardiovascular events.

Cancer

After LT, *de novo* malignancy was reported for 147 patients (44.6%) in the matched cohort. No significant difference was found between addiction follow-up *vs.* control groups regarding incidence of *de novo* malignancy of any type (OR = 1.07; 95% CI, 0.71–1.62; *p* = 0.75). The same result was found in the whole cohort.

Focusing on *de novo* alcohol- and tobacco-related malignancies, which affected 80 patients (24.2%) in the matched cohort, there was no significant difference between addiction follow-up groups *vs.* control groups in incidence (HR 1.36; 95% CI, 0.85–2.16; *p* = 0.21). Only smoking before LT (HR 8.17; 95% CI, 2.57–25.96; *p* = 0.0004) and smoking after LT (HR 2.96; 95% CI,

FLA 5.6.0 DTD ■ JHEPR100832_proof ■ 12 August 2023 ■ 6:28 pm ■ ce

1.88–4.70; p <0.001) were significantly different between both groups. *De novo* alcohol-related malignancy developed among 41 patients (6.7%) but did not show a significant difference either between both groups (HR 0.96; 95% CI, 0.51–1.82; p = 0.91).

There was recurrence of HCC after LT for 21 patients (13.9%) in the matched cohort, but univariate logistic regression analysis did not find a significant difference between the addiction follow-up groups and control groups in incidence (OR 1.17; 95% CI, 0.39–3.47; p = 0.78).

Survival

The mean follow-up time after LT was 9.5 years (0.5–20.4) for the whole cohort, 6.7 years (0.5–12) for the addiction follow-up group, and 11 years (0.5–20.4) for the control group. The survival rate of the whole cohort was 97.4% after 1 year, 83.7% after 5 years, and 70.0% after 10 years. A total of 236 deaths occurred during the follow-up period. The causes of death are listed in Table 5. Kaplan–Meier survival analysis revealed no significant differences between the control group and the addiction follow-up group.

Discussion

In our study, addiction monitoring resulted in a reduction in severe alcohol relapse after LT. Among the 611 liver recipients for ALD surviving over 6 months after hospital discharge, 190 patients underwent both pre- and post-LT standardised addiction care programmes within the Montpellier LT unit. These patients were younger and had a shorter duration of alcohol abstinence before LT compared with matched control patients without any addiction management. Despite the fact that younger age and short duration of abstinence before LT are well-known risk factors for return to alcohol use after LT,[8,12–15] the addiction follow-up group showed a reduction in severe alcohol relapse compared with the control group (7.3% for the addiction follow-up group *vs.* 15.2% for the control group).

These results are consistent with the few previous studies in the literature.[19,20] Given severe alcohol relapse after LT is an issue because of its negative impact on long-term survival as a result of the development of *de novo* malignancies, the onset of cardiovascular diseases, and RAC,[10,25–27] we could expect reducing post-LT relapse to also reduce the risk of all these aforementioned events. The risk factors found associated with severe alcohol relapse in our study (single, abstinence for <6 months, and younger age) correlate with data from the literature, confirming the value of proposing addiction monitoring in young people with a pre-LT abstinence period of <6 months.

ALD occurs in relation to AUD, a behavioural disorder defined by the DSM-V[28] as the presence of at least two of 11 criteria describing physical and psychological dependence, tolerance, as well as clinically and functionally significant impairment. AUD is considered a brain disease.[29] This chronic disease requires a transdisciplinary approach that includes medical treatment and behavioural interventions. An independent, confidential, and specialised addiction management enables for focus on the chronic brain disease.[29,30] Specific pharmacological and non-pharmacological interventions are proposed and adapted to the situation. The LT patient is thus financial support in their chronic addictive disease and is not hampered by the denial that could arise regarding his ‘saviour’, the transplant surgeon.[31]

LT is associated with an improvement in overall quality of life (QOL).[32] This also seems to be the case regardless of the aetiology of the cirrhosis.[33] However, some studies have suggested a lower QOL for patients with ALD, notably because of a poorer professional reintegration after transplantation, a factor known to be associated with QOL.[34] By analogy with the protective effect of abstinence maintenance in AUD,[35] we can hypothesise that prevention of severe alcohol relapse would improve QOL in transplant patients. Nonetheless, this theory has not yet been demonstrated.

One of the main results of our study was the reduction in the number of severe cardiovascular events following addiction follow up (13.1% for the addiction follow-up group *vs.* 22.3% for the control group), even if different follow-up times had to be taken into account for the two groups. ALD recipients are more prone to the development of diabetes during the pre-LT period and to the occurrence of post-LT cardiovascular events. Cardiovascular events are responsible for a high majority of mortality in ALD recipients.[36,37] Given the loss of control over alcohol consumption is often accompanied by smoking, we suggest that reducing severe alcohol relapse could also decrease the number of cardiovascular events.

It should be noted that the proportion of patients identified as post-LT smokers was similar to the proportion of patients identified as pre-LT smokers, both in the addiction follow-up group and in the control group (addiction follow-up group: 41.1% *vs.* 44.2%; control group: 29.5% *vs.* 35.9%). This is taking into account the fact that smoking (active or former) before LT concerned 83.0% of the addiction follow-up group *vs.* 73.3% for the control group. Our intervention here was not sufficient to significantly decrease post-LT smoking, albeit it being part of the addiction programme. Given the potential benefits of smoking cessation for improving long-term survival, reducing occurrence or recurrence of HCC, *de novo* malignancy, and sepsis,[38–43] special efforts regarding smoking should be undertaken. In the previously cited studies, tobacco and alcohol consumption were, individually and/or combined, risk factors for cancer in liver recipients. When patients are exposed to both substances, tobacco and alcohol consumption have a synergistic effect on the development of oral, pharyngeal, laryngeal, oesophageal, and upper airway tumours in the general population.[9,43] However, the reduced severe alcohol relapse in our study did not result in a decrease in the number of *de novo* malignancies developed after LT.

Study limitations

In France, as illustrated herein, the use of integrated addiction specialists within LT units in the context of ALD was/is very rare. Addiction monitoring can range from total standardised integration in the LT unit (the case for Montpellier here), to the absence of a dedicated care pathway, or the use of addiction specialists outside the LT unit. This latter case is the most widespread at present. To perform a robust evaluation of the potential effects of addiction follow up on patient prognosis, we voluntarily chose to evaluate the most integrated form of addiction monitoring; integrated addiction specialists within the LT unit. The current rarity of this care pathway is the reason behind our monocentric inclusion of patients with integrated addiction follow up. Aware that this monocentric aspect could have resulted in study limitations and to avoid confusion bias, we made comparisons to centres that do not systematically use an addiction specialist rather than centres that refer some patients to addiction specialists; moreover, the transplantation periods as well as the follow-up times were not equal between the two groups. However, the groups remain comparable because the

UNCORRECTED PROOF

surgical techniques and the immunosuppression have not been modified for many years.

Finally, the control group is very heterogeneous compared with the study group – it comes from different centres and different time periods. There is a strong possibility of centre effect, which may be a limitation of this study which is related to the retrospective method used. This study was designed as a proof of concept for more ambitious prospective, multicentre studies underway at the Montpellier liver transplant centre.

In conclusion, the present study is the first multicentre study showing a benefit of integrated addiction follow-up on the rate of severe alcohol relapse and on the number of cardiovascular events after LT for patients with ALD. Despite slightly different transplantation time periods and a monocentric inclusion for addiction follow up, we used a rigorous methodology to constitute two comparable groups; ALD recipients with integrated LT unit addiction follow up *vs.* ALD recipients without addiction monitoring. Specific addiction care protocols need to be more standardised among LT units for further evaluation of benefits.

UNCORRECTED PROOF

Abbreviations

ALD, alcohol-associated liver disease; AUD, alcohol-use disorder; ESLD, end-stage liver disease; HCC, hepatocellular carcinoma; HR, hazard ratio; LT, liver transplantation; MELD, model for end-stage liver disease; OR, odds ratio; PSM, propensity score matching; QOL, quality of life; RAC, recurrent alcoholic cirrhosis.

Financial support

No financial support was received for this study.

Conflicts of interest

The authors declare they have no conflicts of interest.

Please refer to the accompanying ICMJE disclosure forms for further details.

Authors' contributions

Concept and design: HD, GPP, JDa, JDu. Experiments and procedures: JDa, GPP, ADB, JUB, MM, LM, SF, FN, CM. Data analysis: LG, NM. Reviewed the literature and wrote the manuscript: HD, Jda. Revised the manuscript: JD, GPP, ADB, FN, SF, MM, JUB, LM, CM, OB.

Data availability statement

The data that support the findings of this study are available from the corresponding author, HDR, upon reasonable request.

Acknowledgements

We thank Jackie Butterworth for his proofreading of the English language, and we also thank all the patients who participated in this study.

Supplementary data

Supplementary data to this article can be found online at https://doi.org/10.1016/j.jhepr.2023.100832.

References

[1] Kwong AJ, Kim WR, Lake JR, Smith JM, Schladt DP, Skeans MA, et al. OPTN/SRTR 2019 annual data report: liver. Am J Transpl 2021;21(Suppl. 2):208–315.
[2] Mathurin P, Lucey MR. Liver transplantation in patients with alcohol-related liver disease: current status and future directions. Gastroenterol Hepatol 2020;5:507–514.
[3] Lucey MR. Liver transplantation for alcoholic liver disease. Nat Rev Gastroenterol Hepatol 2014;11:300–307.
[4] Faure S, Herrero A, Jung B, Duny Y, Daures J-P, Mura T, et al. Excessive alcohol consumption after liver transplantation impacts on long-term survival, whatever the primary indication. J Hepatol 2012;57:306–312.
[5] DiMartini A, Day N, Dew MA, Javed L, Fitzgerald MG, Jain A, et al. Alcohol consumption patterns and predictors of use following liver transplantation for alcoholic liver disease. Liver Transpl 2006;12:813–820.
[6] Cuadrado A, Fábrega E, Casafont F, Pons-Romero F. Alcohol recidivism impairs long-term patient survival after orthotopic liver transplantation for alcoholic liver disease: alcohol recidivism and patient survival. Liver Transpl 2005;11:420–426.
[7] DiMartini A, Dew MA, Day N, Fitzgerald MG, Jones BL, deVera ME, et al. Trajectories of alcohol consumption following liver transplantation. Am J Transpl 2010;10:2305–2312.
[8] De Gottardi A, Spahr L, Gelez P, Morard I, Mentha G, Guillaud O, et al. A simple score for predicting alcohol relapse after liver transplantation: results from 387 patients over 15 years. Arch Intern Med 2007;167:1183–1188.
[9] Dumortier J, Guillaud O, Adham M, Boucaud C, Delafosse B, Bouffard Y, et al. Negative impact of de novo malignancies rather than alcohol relapse on survival after liver transplantation for alcoholic cirrhosis: a retrospective analysis of 305 patients in a single center. Am J Gastroenterol 2007;102:1032–1041.
[10] Dumortier J, Dharancy S, Cannesson A, Lassailly G, Rolland B, Pruvot F-R, et al. Recurrent alcoholic cirrhosis in severe alcoholic relapse after liver transplantation: a frequent and serious complication. Am J Gastroenterol 2015;110:1160–1166.
[11] Watt KDS, Pedersen RA, Kremers WK, Heimbach JK, Sanchez W, Gores GJ. Long-term probability of and mortality from de novo malignancy after liver transplantation. Gastroenterology 2009;137:2010–2017.
[12] Chuncharunee L, Yamashiki N, Thakkinstian A, Sobhonslidsuk A. Alcohol relapse and its predictors after liver transplantation for alcoholic liver disease: a systematic review and meta-analysis. BMC Gastroenterol 2019;19:150.
[13] Jauhar S, Talwalkar JA, Schneekloth T, Jowsey S, Wiesner RH, Menon KVN. Analysis of factors that predict alcohol relapse following liver transplantation. Liver Transpl 2004;10:408–411.
[14] Foster PF, Fabrega F, Karademir S, Sankary HN, Mital D, Williams JW. Prediction of abstinence from ethanol in alcoholic recipients following liver transplantation. Hepatology 1997;25:1469–1477.
[15] Pfitzmann R, Schwenzer J, Rayes N, Seehofer D, Neuhaus R, Nüssler NC. Long-term survival and predictors of relapse after orthotopic liver transplantation for alcoholic liver disease. Liver Transpl 2007;13:197–205.
[16] Dom G, Peuskens H. Addiction specialist's role in liver transplantation procedures for alcoholic liver disease. World J Hepatol 2015;7(18):2091–2099.
[17] Björnsson E, Olsson J, Rydell A, Fredriksson K, Eriksson C, Sjöberg C, et al. Long-term follow-up of patients with alcoholic liver disease after liver transplantation in Sweden: impact of structured management on recidivism. Scand J Gastroenterol 2005;40:206–216.
[18] Addolorato G, Mirijello A, Leggio L, Ferrulli A, D'Angelo C, Vassallo G, et al. Liver transplantation in alcoholic patients: impact of an alcohol addiction unit within a liver transplant center. Alcohol Clin Exp Res 2013;37:1601–1608.
[19] Donnadieu-Rigole H, Jaubert L, Ursic-Bedoya J, Hanslik B, Mura T, Gamon L, et al. Integration of an addiction team in a liver transplantation center. Liver Transpl 2019;25:1611–1619.
[20] Crabb DW, Im GY, Szabo G, Mellinger JL, Lucey MR. Diagnosis and treatment of alcohol-associated liver diseases: 2019 practice guidance from the American association for the study of liver diseases. Hepatology 2020;71:306–333.
[21] European Association for the Study of the Liver. EASL clinical practice guidelines: management of alcohol-related liver disease. J Hepatol 2018;69:154–158.
[22] Arab PA, Izzy M, Leggio L, Bataller R, Shah VH. Management of alcohol use disorder in patients with cirrhosis and liver transplantation. Nat Rev Gasttroenterol Hepatol 2022;19:45–59.
[23] Starosta AN, Leeman RF, Volpicelli JR. The BRENDA model: integrating psychosocial treatment and pharmacotherapy for the treatment of alcohol use disorders. J Psychiatr Pract 2006;12:80–89.
[24] Reus VI, et al. The American Psychiatric Association practice guideline for the pharmacological treatment of patients with alcohol use disorder. Am J Psychiatry 2017;175:86–90.

JHEP|Reports

[25] Conjeevaram HS, Hart J, Lissoos TW, Schiano TD, Dasgupta K, Befeler AS, et al. Rapidly progressive liver injury and fatal alcoholic hepatitis occurring after liver transplantation in alcoholic patients. Transplantation 1999;67:1562–1568. 27.
[26] Saigal S. Evidence of differential risk for posttransplantation malignancy based on pretransplantation cause in patients undergoing liver transplantation. Liver Transpl 2002;8:482–487.
[27] Ursic-Bedoya J, Donnadieu-Rigole H, Faure S, Pageaux G-P. Alcohol use and smoking after liver transplantation; complications and prevention. Best Pract Res Clin Gastroenterol 2017;31:181–185.
[28] American Psychiatric Association. Diagnostic and statistical manual of mental disorders. 5th ed. New York: American Psychiatric Association; 2013.
[29] Volkow ND, Koob GF, McLellan AT. Neurobiologic advances from the brain disease model of addiction. N Engl J Med 2016;374:363–371.
[30] Dom G. Confidentiality and the role of the addiction specialist team in liver transplantation procedures. Commentary on Donnadieu-Rigole *et al*. 2017, Follow-up of alcohol consumption after liver transplantation: interest of an addiction team? Alcoholic Clin Exp Res 2017;41:504–506.
[31] Weinrieb RM, Van Horn DH, McLellan AT, Lucey MR. Interpreting the significance of drinking by alcohol-dependent liver transplant patients: fostering candor is the key to recovery. Liver Transpl 2000;6:769–776.
[32] Cristin DJ, Forman LM, Jackson WE. Beyond survival: targeting health-related quality of life outcomes after liver transplantation. Clin Liver Dis (Hoboken) 2021;17:359–364.
[33] Ruppert K, Kuo S, DiMartini A, Balan V. In a 12-year study, sustainability of quality-of-life benefits after liver transplantation varies with pre-transplantation diagnosis. Gastroenterology 2010;139:1619–1629.e4.
[34] Huda A, Newcomer R, Harrington C, Blegen MG, Keeffe EB. High rate of unemployment after liver transplantation: analysis of the United Network for Organ Sharing database: high unemployment after liver transplantation. Liver Transpl 2012;18:89–99.
[35] Macfarlane VFH, Prentice DA, Walsh MS. The Auckland alcohol detoxification outcome study: measuring changes in quality of life in individuals completing a medicated withdrawal from alcohol in a detoxification unit. Drug Alcohol Depend 2019;202:156–161.
[36] Fatourou EM, Tsochatzis EA. Management of metabolic syndrome and cardiovascular risk after liver transplantation. Lancet Gastroenterol Hepatol 2019;4:731–741.
[37] Watt KDS, Pedersen RA, Kremers WK, Heimbach JK, Charlton MR. Evolution of causes and risk factors for mortality post-liver transplant: results of the NIDDK long-term follow-up study: long-term mortality post-liver transplant. Am J Transpl 2010;10:1420–1427.
[38] Kodali S, Kaif M, Tariq R, Singal AK. Alcohol relapse after liver transplantation for alcoholic cirrhosis—impact on liver graft and patient survival: a meta-analysis. Alcohol Alcohol 2018;53:166–172.
[39] Leithead JA, Ferguson JW, Hayes PC. Smoking-related morbidity and mortality following liver transplantation. Liver Transpl 2008;14:1159–1164.
[40] Mangus RS, Fridell JA, Kubal CA, Loeffler AL, Krause AA, Bell JA, et al. Worse long-term patient survival and higher cancer rates in liver transplant recipients with a history of smoking. Transplantation 2015;99:1862–1868.
[41] Herrero JI, Lorenzo M, Quiroga J, et al. De Novo neoplasia after liver transplantation: an analysis of risk factors and influence on survival. Liver Transpl 2005;11:89–97.
[42] Herrero JI, Pardo F, D'Avola D, Alegre F, Rotellar F, Iñarrairaegui M, et al. Risk factors of lung, head and neck, esophageal, and kidney and urinary tract carcinomas after liver transplantation: the effect of smoking withdrawal: malignancy after smoking withdrawal in LT. Liver Transpl 2011;17:402–408.
[43] Carenco C, Faure S, Herrero A, Assenat E, Duny Y, Danan G, et al. Incidence of solid organ cancers after liver transplantation: comparison with regional cancer incidence rates and risk factors. Liver Int 2015;35:1748–1755.

UNCORRECTED PROOF

Journal of Hepatology, Volume ■

Supplemental information

Integrating an addiction team into the management of patients transplanted for alcohol-associated liver disease reduces the risk of severe relapse

Jules Daniel, Jérôme Dumortier, Arnaud Del Bello, Lucie Gamon, Nicolas Molinari, Stéphanie Faure, Magdalena Meszaros, José Ursic-Bedoya, Lucy Meunier, Clément Monet, Francis Navarro, Olivier Boillot, Georges-Philippe Pageaux, and Hélène Donnadieu-Rigole

Supplementary materials

Integrating an addiction team into the management of patients transplanted for alcohol-associated liver disease reduces the risk of severe relapse

Jules Daniel, Jérôme Dumortier, Arnaud Del Bello, Lucie Gamon, Nicolas Molinari, Stéphanie Faure, Magdalena Meszaros, José Ursic-Bedoya, Lucy Meunier, Clément Monet, Francis Navarro, Olivier Boillot, Georges-Philippe Pageaux, Hélène Donnadieu-Rigole

Supplementary data:

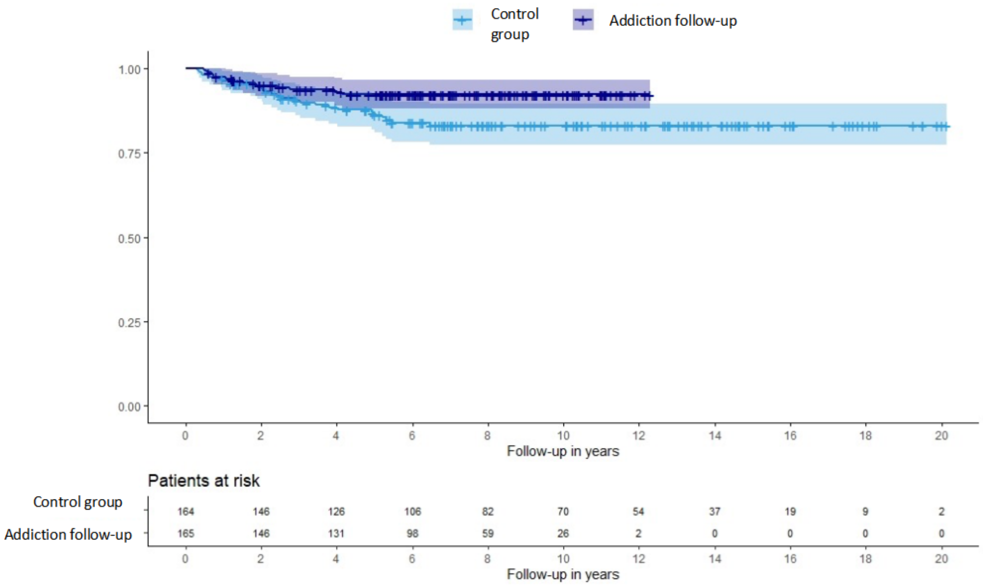

Supplement: Multimedia component 5 [file mmc5.pdf]
